# Malignant Disease of the Humerus

**Published:** 1844-06-01

**Authors:** 


### MALIGNANT DISEASE OF THE HUMERUS.

Mr. Newnham has published in the *Provincial Medical Journal* the case of a young man, seventeen years of age, in whom the disease occurred consequent on an injury received while playing with his sisters. Mr. Newnham, who saw him at that time, considered the accident to be merely an undue stretching, or perhaps a rupture, of some of the fibres of the deltoid, and prescribed accordingly, but some months afterwards the lad was taken to see a surgeon of eminence in town, in consequence of the arm being powerless, and painful when placed in certain positions, and also because any shock communicated to the arm gave great pain. The opinion then expressed was that there was a fracture of the neck of the bone, the surgeon judging from the round orbicular projection of the deltoid, the pulling or drawing forwards and inwards of the shaft, giving a remarkable width to the joint, the perfect facility of communicated motion in all directions, the pain of overhand motions, and the patient's incapacity of performing them but by a jerk from the trunk, and never without pain, while the plainness of the fissure, the pain on pressure, and the very perceptible crepitus, left no doubt on his mind as to the existence of fracture. The limb was accordingly put upon splints, and three weeks afterwards, on a renewed examination, the surgeon came to the conclusion that the process of union had firmly commenced, and that the fissure was considerably less perceptible. With this opinion Mr. Newnham did not coincide. He was fully of opinion that there was not any fracture, and the result justified him in his belief. The disease meanwhile made progress; the splints were of necessity removed, on account of inflammatory action, which had supervened, on the subsidence of which considerable enlargement of the upper part of the bone, attended with exquisite pain, was detected. Fluctuation afterwards becoming evident, a fine lancet was inserted, but only a few drops of intensely black blood came away. The suspicions previously entertained of the existence of malignant disease, were now confirmed by a consultation, at which amputation was decided against, from the situation and extensive connexions of the disease. The treatment employed did not seem to exert any influence on the disease; the tumour continued to increase daily, fungoid growths sprung up through the opening that had been made, and through spontaneous ulcerations, and finally, the poor fellow died, utterly worn out by pain and exhaustion. When the body was examined after death, there was a large swelling of the right shoulder and upper arm, measuring thirty-two inches in its largest circumference, from which protruded three fungoid excrescences. The tumour, which had been tense and firm, had become soft, and evidently contained much fluid matter, which was evacuated partly by puncture, and partly by being ladled out by a teacup; in this way about six pints of a thickish dark-coloured grumous matter were removed; as also three small and undefined portions of bone, not yet thoroughly decomposed. In this fluid matter was also found in a state of ulceration, the cartilaginous structure which had originally formed the covering of the head of the humerus in the glenoid cavity; no trace of the head itself was discernible, nor were there any traces of muscle; the remaining more solid mass consisted of that kind of encephaloid degeneration which is usually found in this species of malignant tumour. Two thirds of the shaft of the humerus were gone, and of the remaining third one-half was in a rapidly advancing state of decomposition. Of the scapula, the acromion process had been consumed, the coracoid partially so; the first, second, and third ribs were deprived of their coverings, and were perfectly rough and scabrous. About two-thirds of the clavicle were also involved in destruction. Encephaloid tubera were found in the lungs, liver, mesenteric glands, and kidneys. The head was not examined. —*Ibid.*

